# A monovalent ion in the DNA binding interface of the eukaryotic junction-resolving enzyme GEN1

**DOI:** 10.1093/nar/gky863

**Published:** 2018-09-24

**Authors:** Yijin Liu, Alasdair DJ Freeman, Anne-Cécile Déclais, David M J Lilley

**Affiliations:** Cancer Research UK Nucleic Acid Structure Research Group, MSI/WTB Complex, The University of Dundee, Dow Street, Dundee DD1 5EH, UK

## Abstract

GEN1 is a member of the FEN/EXO family of structure-selective nucleases that cleave 1 nt 3′ to a variety of branchpoints. For each, the H2TH motif binds a monovalent ion and plays an important role in binding one helical arm of the substrates. We investigate here the importance of this metal ion on substrate specificity and GEN1 structure. In the presence of K^+^ ions the substrate specificity is wider than in Na^+^, yet four-way junctions remain the preferred substrate. In a combination of K^+^ and Mg^2+^ second strand cleavage is accelerated 17-fold, ensuring bilateral cleavage of the junction. We have solved crystal structures of *Chaetomium thermophilum* GEN1 with Cs^+^, K^+^ and Na^+^ bound. With bound Cs^+^ the loop of the H2TH motif extends toward the active site so that D199 coordinates a Mg^2+^, buttressed by an interaction of the adjacent Y200. With the lighter ions bound the H2TH loop changes conformation and retracts away from the active site. We hypothesize this conformational change might play a role in second strand cleavage acceleration.

## INTRODUCTION

The Holliday junction (1) is the central intermediate in homologous genetic recombination, that is important in meiotic chromosome segregation and DNA repair in mitotic cells. The junction comprises four helices connected by the continuity of the component strands (a 4H junction (2)), and must be processed in some manner. There are two distinct ways this is achieved in eukaryotic cells, dissolution and resolution. In dissolution two four-way junctions are translocated toward each other by the action of BLM helicase, and then disconnected by topoisomerase IIIα (3–5). Alternatively junctions may be processed by resolution, requiring the action of a junction-resolving enzyme.

Junction-resolving enzymes are nucleases that bind selectively to four-way DNA junctions and make bilateral cleavages (6). Two main junction-resolving enzymes have been identified in eukaryotes. The SLX1-SLX4-Mus81-Eme1 enzyme is part of a larger complex of proteins organized by the SLX4 scaffold (7–11), in which individual cleavages are introduced by the SLX1 and Mus81 nucleases (12–14). The other junction-resolving activity is GEN1, originally isolated by biochemical fractionation of HeLa cell extracts by West and colleagues (15). Paralogs were also identified in yeast (called Yen1) (15) and *Caenorhabditis elegans* (16). Unfortunately human GEN1 is very prone to aggregation and so not well suited to structural and biophysical work, but we found that the paralog from the thermophilic fungus *Chaetomium thermophilum* (termed *Ct*GEN1) had excellent properties enabling its full biochemical characterization (17). We crystallized *Ct*GEN1 as the complex with its DNA product (18). A structure of the human paralog has also been presented, as a non-specific complex with a DNA duplex in the site of the non-cleaved duplex (19). SLX4 and GEN1 exhibit synthetic lethality resulting from unprocessed Holliday junctions leading to dysfunctional mitosis (20,21). Furthermore Yen1 is required to resolve persistent junctions during meiosis in a Δ*mus81* cell (22).

GEN1 is a member of the family of structure-selective nucleases that target various branched DNA molecules (23), that include FEN1 (24–26), EXO1 (27) and XPG (28). These enzymes hydrolyze a phosphodiester linkage 1 nt 3′ to a variety of branchpoints, including flaps, i.e. 5′ single stranded sections (FEN1), a 3′ single-stranded overhang or nick (EXO1) and single-stranded bubbles (XPG or Rad2). Crystal structures for DNA complexes of all four nucleases have been solved (18,19,29–31), and each share a common core architecture (Supplementary Figure S1) comprising a mixed β-sheet flanked on both sides by α-helices, and an active center in which two divalent metal ions are bound by a number of carboxylate side chains. FEN1, especially those of phages T4 (32) and T5 (33) represents the basic core structure, and the other members of the family are elaborated by additional extensions or insertions. FEN1 and EXO1 have a 40-aminoacid helical arch that acts as a gateway to select the single-stranded sections of the substrate (29,30). By contrast GEN1 cleaves DNA 1 nt 3′ to the point of strand exchange on two strands of four-way junctions, and the key distinction from the other members of the family is that it acts as a homodimer (17) and thus makes two incisions into the junction. An important property of junction-resolving enzymes is that the second strand cleavage event is generally accelerated relative to the first (17,34,35), helping to ensure that a complete resolution of the junction is achieved.

FEN1 and GEN1 bind two connected DNA helices in a near identical manner, where the two helical axes are close to perpendicular in the bound complex, hinged about the connecting phosphodiester linkage on the continuous strand (18,29). An additional chromodomain in *Ct*GEN1 makes extended contacts with a longer second (uncleaved) arm (18), so that all four helical arms in the dimer complex are extensively contacted by the protein. The cleaved arm (i.e. 3′ to the position of scissile phosphate) is bound in a closely similar manner in all four members of the FEN/EXO family. In each case there is a structural feature often called the H2TH that comprises two α helices directed toward the DNA located one turn of helix away from the cleavage site. In FEN1, EXO1 and Rad2 this has been found to be connected by a loop that binds a K^+^ ion (29–31), and extends toward the active site of the nuclease. Our original crystallographic study of *Ct*GEN1 (18) was performed on a complex that was formed in the presence of Na^+^, not K^+^ ions and at that time we could not trace the chain of the connecting loop.

The majority of biochemical analysis of both human (36) and *Ct*GEN1 (17) have also been performed in the presence of Na^+^ as the monovalent cation. These experiments indicated that GEN1 was strongly selective for Holliday junctions. There was some indication of activity on other branched species, but the enzyme was most active on four-way junctions. By contrast, GEN1 from *Drosophila* was found to cleave a wider range of branched substrates (37), and significantly those studies were performed in the presence of K^+^ ions. Given the importance of K^+^ ions in the activity and structure of the other FEN/EXO family members we sought to re-examine both the protein structure and substrate range of *Ct*GEN1 in the presence of other monovalent ions. These differ in ionic size and coordination number from Na^+^ (1.1 Å, hexacoordinate) to Cs^+^ (1.67 Å, octacoordinate), and thus could conceivably affect the conformation of the protein loop. The results presented here show that both the substrate range and the conformation of the H2TH loop are indeed affected by the nature of the bound monovalent ion. Moreover, measurement of strand cleavage under optimal conditions in the presence of K^+^ ions leads to a 17-fold acceleration of second strand cleavage.

## MATERIALS AND METHODS

### Synthesis of DNA oligonucleotides and construction of junction species

Oligonucleotides were synthesized using β-cyanoethyl phosphoramidite chemistry {Beaucage, 1981 #6; Sinha, 1984 #171}. Fully deprotected oligonucleotides were purified by gel electrophoresis in polyacrylamide gels (10–20% (w/v) depending upon oligonucleotide length) in 90 mM Tris.borate (pH 8.5), 2 mM ethylenediaminetetraacetic acid (EDTA) (TBE buffer) containing 8 M urea, and recovered by electroelution and ethanol precipitation. Helical junctions were assembled by mixing stoichiometric quantities of strands (all sequences shown in Supplementary Figure S2 and Table S1), and annealed by incubation in 20 mM Tris–HCl (pH 8), 50 mM NaCl for 5 min at 85°C, followed by slow cooling. These were purified by electrophoresis under non-denaturing conditions in 8% polyacrylamide in TBE buffer for 8 h at 20°C, and recovered by electroelution and ethanol precipitation.

### Preparation of *Ct*GEN1

A construct expressing *Ct*GEN1 1–487 with a C-terminal six-histidine tag was cloned into a pWaldo vector (18). After confirming the correct sequence the plasmid was transformed and expressed in *Escherichia coli* Rosetta (DE3) competent cells (Novagen). *Ct*GEN1 was purified by successive chromatography steps based using Ni-NTA affinity, heparin, gel filtration and Mono S ion exchange. Purified *Ct*GEN1 migrated as a single band on an overloaded polyacrylamide gel in the presence of sodium dodecyl sulphate. *Ct*GEN1 concentration was estimated from absorbance at 280 nm using A_280_ = 50600 M^−1^ cm^−1^.

### Analysis of cleavage kinetics of a four-way DNA junction by *Ct*GEN1

Cleavage kinetics were analyzed under single-turnover conditions. 200 nM *Ct*GEN1 was incubated with 2–5 nM DNA junction radioactively [5′-^32^P]-labeled on one strand in 10 mM cacodylate (pH 6.5), 50 mM NaCl or KCl, 0.1% bovine serum albumin (BSA) for 3 min at 37°C. Cleavage was initiated by addition of 1 mM MgCl_2_ and aliquots were removed at chosen times and EDTA added to a final concentration of 50 mM to terminate the reaction. One volume of 90% formamide was added and the samples were then denatured at 90°C for 15 min before separation of substrates and products by electrophoresis in a 15% (19:1) polyacrylamide gel in TBE containing 8 M urea at 80 W. Gels were dried and exposed to storage phosphor screens and quantified using a Fuji BAS 1500 phosphorimager using MacBAS software. The fraction of DNA cleaved at time *t* (*f*_t_) was fitted by nonlinear regression analysis to the equation:

(1) \begin{equation*}{f_{\rm{t}}} = {f_{\rm{f}}}.\left( {1 - {\rm{exp}}\left( { - {k_{\rm{c}}}t} \right)} \right)\end{equation*}

where *f*_f_ is the fraction of DNA cleaved at the end of the reaction and *k*_c_ the rate of cleavage. All numerical data in this work were fitted by non-linear regression using KaleidaGraph 4.5.3 (Synergy Software).

### Analysis of binding affinity

8 μl of 0.2 –1 nM radioactively [5′-^32^P]-labeled DNA in 10 mM Tris (pH7.5), 0.1% BSA and 100 mM KCl or NaCl were mixed with serial dilutions of 8 μl of *Ct*GEN1 in 10 mM Tris (pH 7.5), 100 mM NDSB-195 (Affymetrix), 0.1% BSA and 1 mM EDTA and incubated for 20 min at 20°C. After the addition of Ficoll-400 to 2.5%, the samples were loaded onto a 6 or 10% (29:1) polyacrylamide gel in TBE and subjected to electrophoresis for 1.5–2.5 h. Gels were dried onto Whatman 3MM paper and free and bound DNA quantified by autoradiography. Plots of fraction DNA bound (*f*_b_) as a function of *Ct*GEN1 concentration were fitted to the Hill equation for cooperative binding:

(2) \begin{equation*}{f_{\rm{b}}} = {P_{\rm{t}}}^{\rm{n}}/\left( {{P_{\rm{t}}}^{\rm{n}} + {K_{\rm{d}}}} \right)\end{equation*}

where P_t_ is the total protein concentration and *n* the Hill coefficient. From this the dissociation constant *K*_d_ and *n* were determined.

### Kinetic analysis of cruciform cleavage

The cruciform-containing plasmid pHRX3 was generated by ligating the oligonucleotides 5′ GAATTCGCAGCCTGAGCGATATATATATATATATATATATATCGCTCAACTCAGATCCTCT and 5′ CTTAAGCGTCGGACTCGCTATATATATATATATATATATATAGCGAGTTGAGTCTAGGAGA between the EcoRI and BamHI sites of pAT153 (Supplementary Figure S3). The central alternating (AT)_12_ repeated sequence ensures facile extrusion of the cruciform in negatively supercoiled DNA (38). This was transformed into *E. coli* DH5α and grown to A_600_ = 0.6 at 37°C. The plasmid was amplified by treatment with 50 μg/ml chloramphenicol overnight. Plasmid DNA was isolated using Qiagen maxiprep purification kit at 4°C and purified by two rounds of isopynic CsCl/ethidium bromide ultracentrifugation. Supercoiled DNA was recovered by side puncture, ethidium bromide removed by extraction with *n*-butanol and the DNA subjected to extensive dialysis against 20 mM Tris (pH 8.0), 1 mM EDTA to remove CsCl. DNA concentration was measured spectrophotometrically.

10 nM pHRX3 plasmid was pre-incubated with 200 nM *Ct*GEN1 in 10 mM cacodylate (pH 6.0), 50 mM KCl, 0.1% BSA for 3 min at 37°C before the cleavage reaction was initiated by addition of MgCl_2_ or MnCl_2_ to a final concentration 1 mM. Aliquots were removed at specific times and 50 mM EDTA was added to terminate the reaction. A total of 1 μl proteinase K was added and the samples were incubated overnight at room temperature. Supercoiled, linear and nicked DNA were separated by electrophoresis in 1% agarose gels in TBE buffer. Gels were stained with Safeview nucleic acid stain (NBS Biological Ltd), extensively washed with water and scanned using a FLA-2000 fluorescent image analyzer (Fuji) using excitation at 473 nm with an emission filter at 580 nm.

The fractions of supercoiled (S), nicked (N) and linear (L) plasmid as a function of time (t) were fitted to a model (Supplementary Figure S4) in which the first cleavage occurs at a rate *k*_1_, followed by cleavage of the other strand with rate *k*_2_, according to the equations (17):

(3) \begin{equation*}{\left[ {\rm{S}} \right]_{\rm{t}}}/{\left[ {\rm{S}} \right]_{\rm{0}}} = {\rm{exp}}\left( { - {k_{\rm{1}}}{\rm{t}}} \right)\end{equation*}

(4) \begin{equation*}{\left[ {\rm{N}} \right]_{\rm{t}}}/{\left[ {\rm{S}} \right]_{\rm{0}}} = {k_1}/\left( {{k_{\rm{2}}} - {k_{\rm{1}}}} \right).\left( {{\rm{exp}}\left( { - {k_{\rm{1}}}{\rm{t}}} \right) - {\rm{exp}}\left( { - {k_{\rm{2}}}{\rm{t}}} \right)} \right)\end{equation*}

(5) \begin{eqnarray*}{\left[ {\rm{L}} \right]_{\rm{t}}}/{\left[ {\rm{S}} \right]_{\rm{0}}} &=& 1 - {\rm{exp}}\left( { - {k_{\rm{1}}}{\rm{t}}} \right)\nonumber\\ &&- {k_{\rm{1}}}/\left( {{k_{\rm{2}}} - {k_{\rm{1}}}} \right).\left( {{\rm{exp}}\left( { - {k_{\rm{1}}}{\rm{t}}} \right) - {\rm{exp}}\left( { - {k_2}{\rm{t}}} \right)} \right)\end{eqnarray*}

### Crystallographic analysis

DNA used for crystallography was synthesized and purified as above. The four DNA strands were hybridized in 5 mM HEPES (pH 7) 20 mM NaCl with slow cooling from 90°C to room temperature. Purified *Ct*GEN1 was mixed with a four-way DNA junction based on junction 3 comprising 15 bp in each helical arm. Equal volumes of 100 μM DNA and 100 μM protein were mixed in a crystallization buffer containing 100 mM HEPES (pH 7.5), 2 mM MgCl_2_, 20% PEG10000. Hanging drops were suspended above a well containing the same crystallization buffer and subjected to vapor diffusion at 7°C. Cubic-shaped crystals grew up to 0.1 mm in 2 weeks. Exchange of monovalent ions was performed by soaking the crystals in solutions of 100 mM HEPES (pH 7.5), 20% PEG10000 and 10 mM KCl or 10 mM CsCl for 1 h. Crystals were then immersed in 100 mM HEPES (pH 7.5), 20% PEG10000, 30% sucrose as cryoprotectant followed by dehydration by vapor diffusion with saturated KNO_3_ for 60–90 min at 7°C. Finally the crystals were stored under liquid nitrogen.

Crystallographic datasets were collected at Diamond light source and structures were solved by molecular replacement using the initial model PDB ID: 5CNQ using the PHENIX suite (39). The model was adjusted manually and subjected to several rounds of adjustment and optimization. Final models were refined to a resolution of 2.45 Å (Na^+^), 2.4Å (K^+^) and 2.66 Å (Cs^+^), respectively. Crystallographic statistics are presented in Supplementary Table S3. Coordinates for structures in Na^+^ (6GRC), K^+^ (6GRB) and Cs^+^ (6GRD) have been deposited with the PDB.

## RESULTS

### *Ct*CEN1 substrate selectivity as a function of monovalent metal ions

In our original analysis of *Ct*GEN1 we compared the cleavage products generated after incubation with different branched DNA species, i.e. four-way (4H) junctions, nicked three-way junctions (often referred to as a replication fork analog) and splayed helix junctions (a helix with non-complementary 3′ and 5′ single-stranded extensions at one end). After a 1 h incubation in the presence of 50 mM Na^+^ and 10 mM Mg^2+^ ions at pH 7.5 we found that only the four-way junction was significantly cleaved, the other branched species being largely unaffected (17). We therefore repeated this analysis, whereby the Na^+^ ions were exchanged for other monovalent metal cations, particularly K^+^. We have also measured the affinity of *Ct*GEN1 binding to various branched DNA substrates by electrophoretic retardation analysis in the presence of Na^+^ and K^+^ ions.

**Figure 1.**
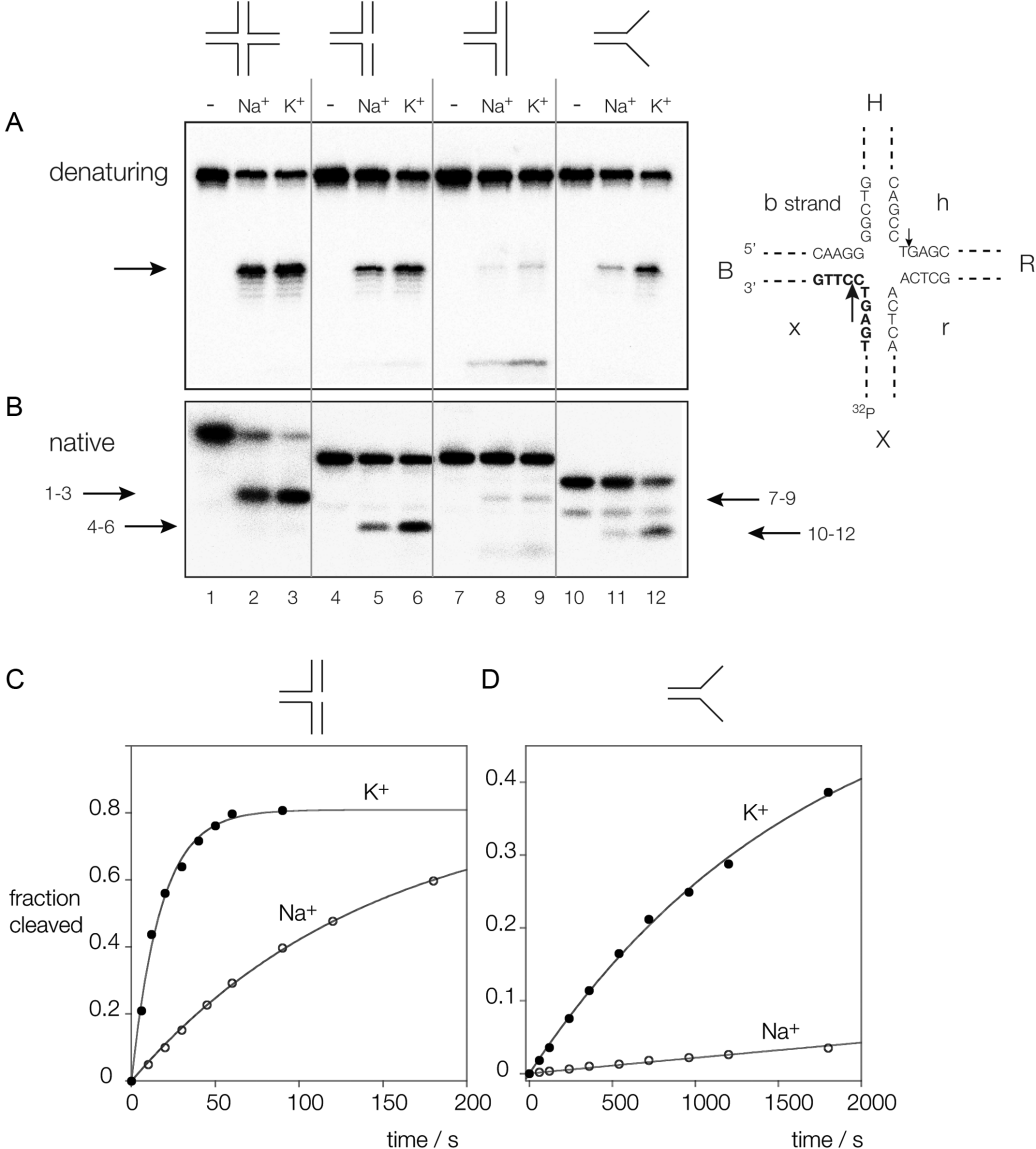
Cleavage of branched DNA substrates by *C*tGEN1 as a function of the monovalent metal ion present. Substrates radioactively [5′-^32^P]-labeled on the x strand were incubated with *Ct*GEN1 in buffer containing 10 mM cacodylate (pH 6.5), 1 mM MgCl_2,_ 50 mM NaCl or KCl, 0.1% BSA for 3 min at 37°C. The products separated by polyacrylamide gel electrophoresis followed by phosphorimaging. (**A**) Denaturing gel electrophoresis. The major product of cleavage is arrowed. Note that each substrate is cleaved at the same position. The scheme on the right shows the sequence of the core of the junction, and the nomenclature of the arms and strands. The position of the radioactive label is indicated and the cleavage sites are arrowed. The major cleavages are made in the B and R arms, 1 nt 3′ to the point of strand exchange on the h and x strands. In this experiment the x strand is radioactively [5′-^32^P]-labeled, and thus only cleavage in this strand is detected. (**B**) Native gel electrophoresis. The products of *Ct*GEN1 cleavage are arrowed, with the relevant tracks indicated; e.g. the arrow labeled 1–3 indicates the product of four-way junction cleavage. In A and B tracks 1–3 contain the four-way junction (4H), tracks 4–6 contain the nicked three-way junction, tracks 7–9 contain the three-way junction (3H) and tracks 10–12 contain the splayed helix junction. For each is shown no cleavage with *Ct*GEN1 i.e. intact junction, tracks 1, 4, 7 and 10, cleavage with *Ct*GEN1 in the presence of Na^+^ ions, tracks 2, 5, 8 and 11, and cleavage with *Ct*GEN1 in the presence of K^+^ ions, tracks 3, 6, 9 and 12. (**C**) Progress curve for cleavage of a nicked three-way junction. (**D**) Progress curve for cleavage of a splayed helix junction. In both C and D cleavage in K^+^ and Na^+^ ions are indicated by closed and open circles, respectively. The data are fitted to single exponential functions (Equation 1; lines). Sequences and secondary structures are presented in Supplementary Figure S2 and Table S1.

Figure 1A and B show the products of incubation of four-way, three-way (3H junction (2)), nicked three-way and splayed helix junctions (the sequences are shown in Supplementary Figure S2 and Table S1) in the presence of either Na^+^ or K^+^ ions, analyzed by separation on polyacrylamide gels under denaturing and native conditions, respectively. While the four-way junction is substantially cleaved in the presence of either monovalent ion after 1 min (Supplementary Figure S5), cleavage of the other branched species is more dependent on the cation present. The three-way junction is almost uncleaved under either condition, but the nicked three-way junction and the splayed helix junctions are cleaved to a significantly greater extent in the presence of K^+^ ions. Each construct contains two strands in common (b and x in the four-way junction) and is radioactively [5′-^32^P]-labeled on the x strand. Electrophoresis of the products under denaturing conditions (Figure 1A) reveals that each of the substrates is cleaved by *Ct*GEN1 at the same position, i.e. 1 nt 3′ to the branchpoint. The rates of cleavage have been determined under single-turnover conditions in the presence of 1 mM Mg^2+^, presented in Supplementary Table S2 and cleavage reaction progress curves in Na^+^ and K^+^ ions for the nicked three-way junction species and the splayed helix are shown in Figure 1C and D. The ratios of cleavage rates in the two ions (*k*_K_/*k*_Na_) were 16 for the splayed helix junction, and 8 for the nicked three-way junction. Although the four-way junction is efficiently cleaved in both ions, the rate is nevertheless affected by the nature of the monovalent ion and is 2-fold faster in the presence of K^+^ ions (Supplementary Figure S5). The affinity of binding *Ct*GEN1 to a four-way junction is also 2-fold higher in K^+^ ions compared to Na^+^ (Supplementary Figure S6 and Table S2).

While the substrate range is broadened in the presence of K^+^ ions, the absolute rates are significantly higher for the four-way junctions than the other species. Thus the four-way junction is cleaved twice as fast as the nicked three-way junction and 180 times faster than the splayed helix species. Cleavage of the three-way junction is too slow to measure. The binding affinity is also significantly higher for the four-way junction compared to that for the three-way or nicked three-way junctions (Supplementary Figure S7 and Table S2). For each species the affinity was higher in K^+^ ions compared to that in Na^+^. Binding to the splayed helix construct did not result in discrete retarded species, but rather diffuse smears that were difficult to quantify. The three-way and nicked three-way junctions make an interesting comparison. *Ct*GEN1 binds to the three-way junction with higher affinity (Supplementary Table S2), yet the nicked species is well cleaved while the three-way junction is not. It is likely that the nicked three-way junction has the flexibility to adopt a similar conformation to the four-way junction when bound to *Ct*GEN1, whereas the three-way junction lacks this flexibility and is therefore not cleaved despite being bound.

The cleavage of the four-way junction has also been analyzed in the presence of Rb^+^ and Cs^+^ ions (Supplementary Table S2). The rate in Rb^+^ ions is very similar to the rate observed in K^+^, while that in Cs^+^ is a little slower.

### Acceleration of second strand cleavage in the presence of K^+^ and Mg^2+^ metal ions

A key difference between GEN1 and the other members of the FEN/EXO family is that GEN1 acts in dimeric form to make bilateral cleavages in a four-way DNA junction. As a class, the junction-resolving enzymes typically cleave the second site faster than the first (17,34,35), helping to achieve a productive resolution of the junction. We have previously shown that there is second strand acceleration of cleavage for *Ct*GEN1, but the factor between first and second strand cleavage rates was relatively modest (17). In the light of the effects of changing the monovalent metal ions we have therefore re-investigated the acceleration of second strand cleavage in the presence of K^+^ ions. This is studied by the cleavage of a cruciform extruded from a negatively supercoiled DNA plasmid (34,35,40). The first cleavage results in the formation of a nicked circle of DNA, and second cleavage within the lifetime of the enzyme–DNA complex linearizes the plasmid (Supplementary Figure S4). Since supercoiled, nicked and linear DNA are well separated by gel electrophoresis in agarose the formation of each species is easily quantified. In this study we used the plasmid pHRX3, the cruciform of which is a good substrate for *Ct*GEN1 (Supplementary Figure S3). Cleavage was studied under two conditions, in the presence of 50 mM K^+^ together with either 1 mM Mn^2+^ or Mg^2+^ ions.

**Figure 2.**
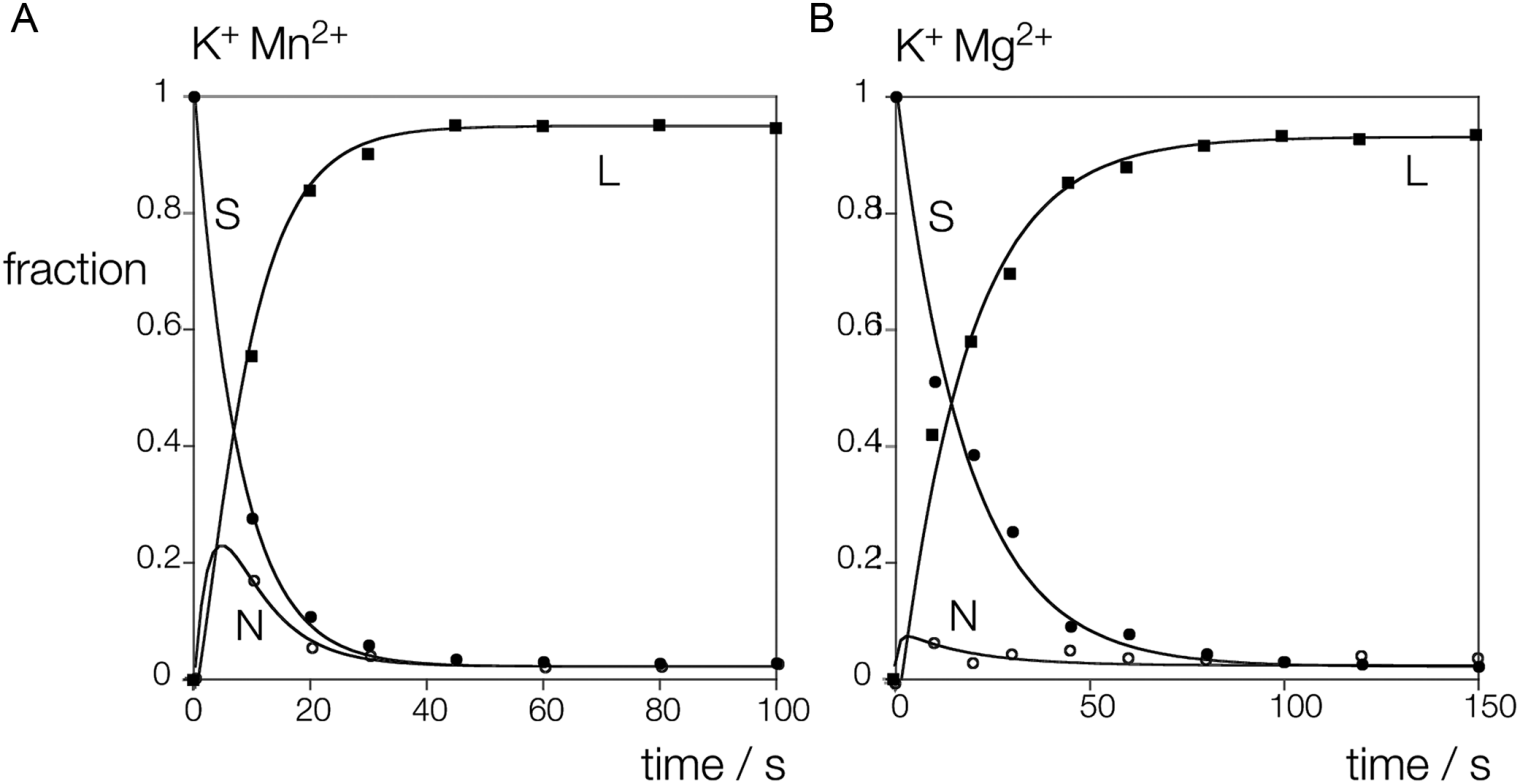
Analysis of first and second strand *Ct*GEN1 cleavage kinetics using a supercoil-stabilized cruciform structure. A scheme showing the principle of the experiment is shown in Supplementary Figure S4, and the sequence of the cruciform is shown in Supplementary Figure S3. (**A**) Cleavage performed in the presence of K^+^, Mn^2+^ ions. (**B**) Cleavage performed in the presence of K^+^, Mg^2+^ ions. 10 nM pHRX3 plasmid was pre-incubated with 200 nM *Ct*GEN1 in 10 mM cacodylate (pH 6.0), 50 mM KCl, 0.1% BSA for 3 min at 37°C before the cleavage reaction was initiated by addition of MgCl_2_ or MnCl_2_ to a final concentration of 1 mM. The data have been plotted and fitted to Equations 3–5. S: supercoiled DNA (closed circles), N: nicked circular DNA (open circles), L: linear DNA (closed squares).

The time dependence of the relative concentrations of supercoiled, nicked and linear DNA are plotted in Figure 2 for the two conditions. In both cases the loss of supercoiled DNA and the generation of linear product are clear, but the major difference between the two datasets is the extent of formation of nicked circular intermediate. In the presence of Mn^2+^ ions there is significant accumulation of nicked species within the first 10 s of reaction, whereas in Mg^2+^ ions this is lower. The data have been fitted to the integrated rate equations for sequential cleavage reactions (17) (Equations 3–5), from which the rate constants for first (*k*_1_) and second (*k*_2_) strand cleavage have been calculated. In 50 mM K^+^, 1 mM Mn^2+^ ions *k*_1_ = 0.13 and *k*_2_ = 0.35 s^−1^, while in 50 mM K^+^, 1 mM Mg^2+^ ions *k*_1_ = 0.056 and *k*_2_ = 0.93 s^−1^. Under both conditions there is an acceleration of second strand cleavage. In 50 mM K^+^, 1 mM Mn^2+^ ions the ratio *k*_2_/ *k*_1_ is 2.6, a relatively modest second strand acceleration. However in the presence of 50 mM K^+^, 1 mM Mg^2+^ ions the ratio *k*_2_/ *k*_1_ rises significantly to 16.9. Thus under physiological conditions there will be a substantial acceleration of second strand cleavage.

### Structural analysis of *Ct*CEN1 with three alternative monovalent metal ions

In the light of the differences in the biochemical properties of *Ct*GEN1 in the presence of different monovalent metal ions we have obtained crystal structures in the presence of three alternative group I metal ions. Wild-type *Ct*GEN1 bound to a four-way junction was crystallized in the presence of Na^+^ and Mg^2+^ ions, resulting in crystals of a product complex because under these conditions *Ct*GEN1 is active. These crystals were then soaked in either K^+^ or Cs^+^ ions to replace the Na^+^ ions. Crystals diffracted to 2.4 Å (K^+^) and 2.66 Å (Cs^+^) resolution, and each structure was solved by molecular replacement using our original structure PDB ID: 5CNQ (18).

**Figure 3.**
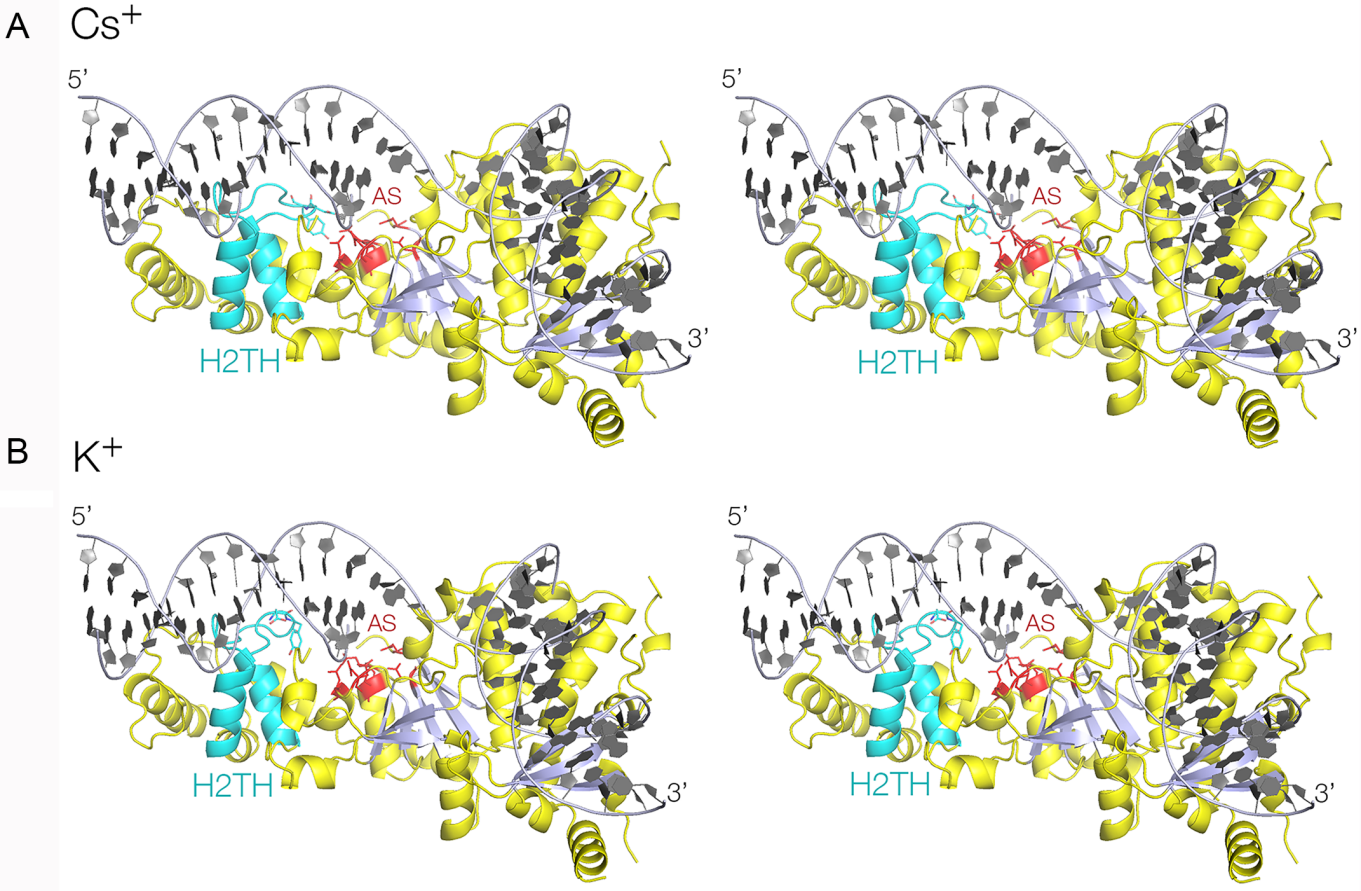
X-ray crystal structures of the functional unit of a complex of *Ct*GEN1 bound to its resolution product. Parallel-eye stereoscopic views are shown. **A**. Structure with a bound Cs^+^ ion. **B**. Structure with a bound K^+^ ion. In both structures the H2TH region is highlighted in cyan, and the active site (AS) residues in red.

Aside from the differences in the H2TH region of the protein discussed below, the overall structures of the *Ct*GEN1 product complex (Figure 3) were closely similar, and superimpose with an RMSD = 0.24 Å (K^+^ versus Na^+^) and RMSD = 0.36 Å (Cs^+^ versus Na^+^). As we have found previously (18), the structure of *Ct*GEN1 contains a central seven-strand β-sheet flanked on both sides by 15 α-helices, with a topology very similar to the other FEN/EXO family of nucleases. In addition there is the C-terminal chromodomain comprising a three-strand antiparallel β-sheet flanked by four α-helices. The protein presents a relatively planar DNA-binding surface on which bind the two mutually perpendicular helical arms of the product.

### Structural analysis of *Ct*CEN1 with a bound Cs^+^ ion

**Figure 4.**
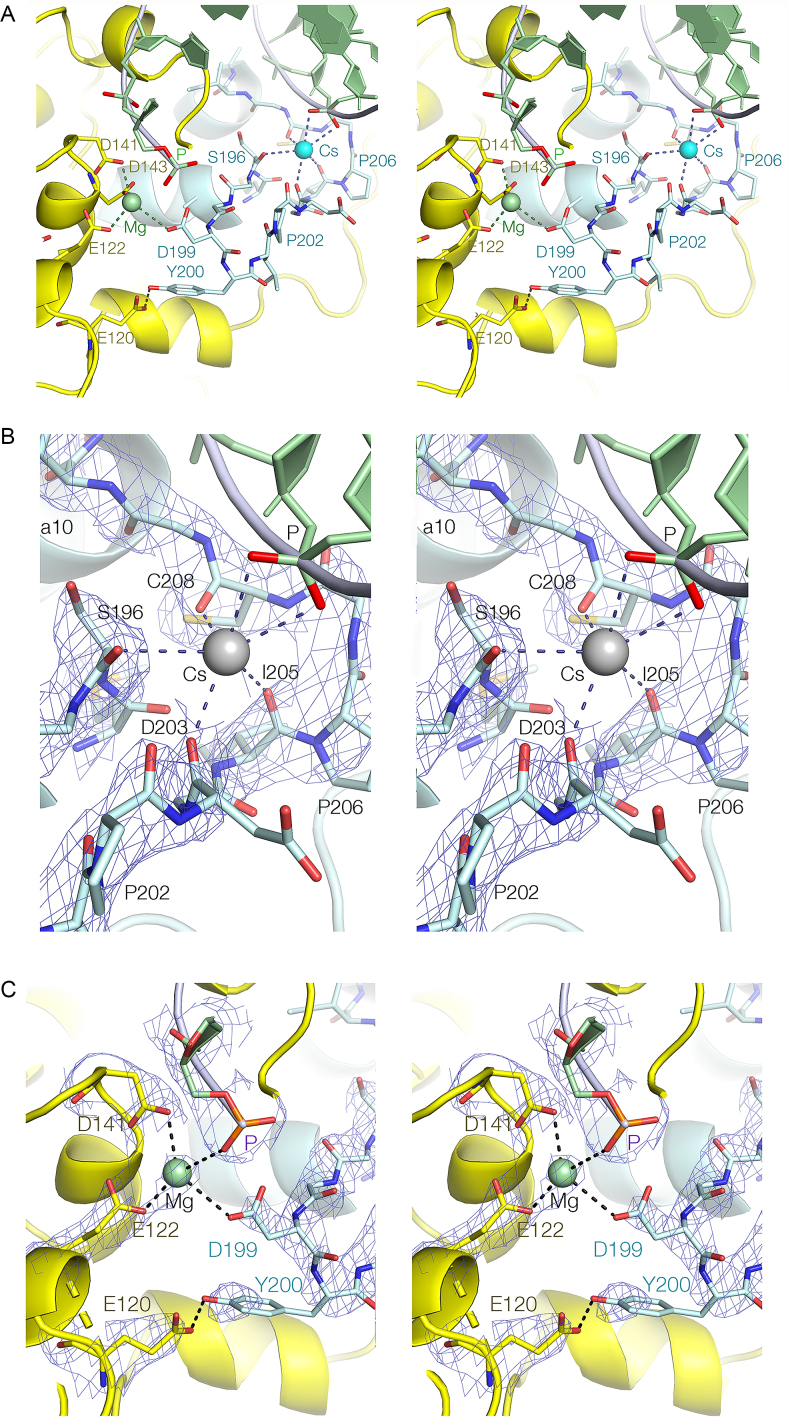
The H2TH region of *Ct*GEN1 bound to its resolution product with a bound Cs^+^ ion. Parallel-eye stereoscopic views are shown. ( **A**) The complete loop (cyan) showing the interaction with the active site. (**B**) The Cs^+^ binding site. (**C**) The interaction of the distal end of the H2TH loop with the active site. The Mg^2+^ ion (Mg, green) is the M2 ion of the active site. B and C show the 2**F**_o_-**F**_c_ electron density map for the key components of the interactions.

Electron density corresponding to the section of polypeptide lying between α-helices 9 and 10, also known as the H2TH region, is clearly visible along with a bound Cs^+^ ion (Figure 4). The section between S196 and I210 forms an extended loop that is 17Å long, with D199 at its apex. The sequence of the loop is quite hydrophobic, and includes two proline, two isoleucine, a leucine and six glycine residues. The Cs^+^ ion is bound at the open end of the loop (Figure 4A), coordinated by oxygen atoms of both the DNA and the peptide. Four main chain O atoms from S196, D203, I205 and C208 form a bowl-like arrangement on one side of the ion. Three (D203, I205 and C208) are located at the C-terminal end of the loop where the backbone adopts an S-shaped trajectory, while the fourth (S196) is at the N-terminal end where the loop connects to α9. On the other side of the metal ion both non-bridging O atoms of the DNA approximately one turn of helix from the cleavage site also make direct contacts. Thus the Cs^+^ ion is coordinated by a total of six O atoms, with an average Cs-O distance of 3.22 Å. At the extreme end of the polypeptide loop the side chain of D199 enters the active site, with its carboxylate group coordinated to the M2 Mg^2+^ ion (Figure 4C). In addition the phenolic O of Y200 donates a hydrogen bond to the carboxylate of E120 at the end of α6. This interaction is highly conserved in the FEN/EXO family (e.g. the equivalent interaction between Y234 and E158 in human FEN1; PDB ID: 3Q8K), and is particularly well defined in the present electron density map. The importance of the interacting amino acids on the activity of *Ct*GEN1 is further explored by mutagenesis as discussed below. The H2TH loop connects the DNA phosphate at the cleavage site with the binding site one helical turn away, potentially coupling the activity of the enzyme to the conformation of the junction.

### Structural analysis of *Ct*CEN1 with a bound K^+^ ion

While the overall structure of the *Ct*GEN1 product complex following a crystal soak with K^+^ ions is closely similar to the Cs^+^-bound structure, the H2TH region between α-helices 9 and 10 takes a significantly different trajectory (Supplementary Figure S8). The C-terminal end of the loop (I205 to G209) adopts the same conformation as that in the Cs^+^ structure (RMSD = 0.21 Å), so that the K^+^ ion is positioned similarly to Cs^+^, and the main chain O atoms of I205 and C208 together with S196 at the N-terminal end bind the K^+^ ion in the same manner. However, the main chain O of D203 is 8.7 Å from the K^+^ metal ion, and this is the major difference between the metal ion coordination in the two structures. The K^+^ ion is coordinated by the two non-bridging O atoms of the DNA phosphate in the same manner as the Cs^+^ ion. The N-terminal section of the loop between G196 and G204 takes a very different trajectory in the K^+^ structure. The effect of this is that the loop is less extended compared to the Cs^+^ structure, thus disrupting the interaction of D203 with the monovalent metal ion. Importantly, D199 is no longer within the active site in the K^+^ structure, and its carboxylate is 12 Å from the M2 Mg^2+^ ion.

### Structural analysis of *Ct*CEN1 with a bound Na^+^ ion

**Figure 5.**
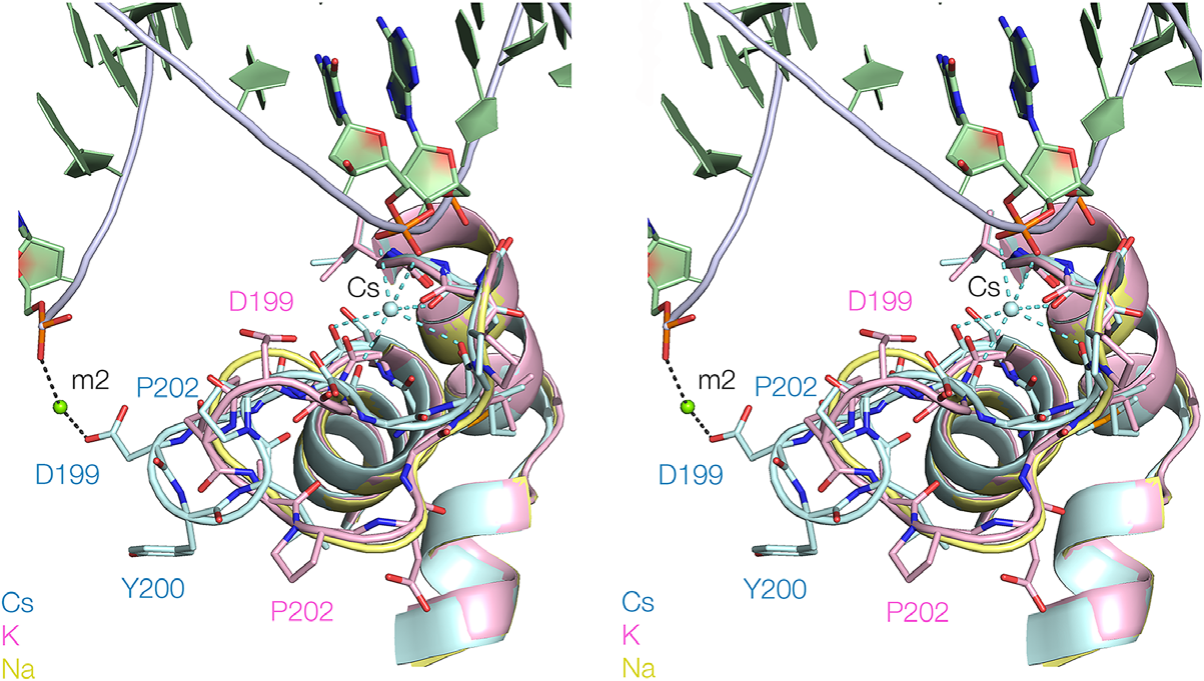
A superposition of the H2TH regions of *Ct*GEN1 with bound Cs^+^, K^+^ and Na^+^. The protein structures are shown in cyan, pink and yellow, respectively. DNA is shown in green. The Cs^+^ ion is shown as a sphere. The paths of the loops are shown by ribbons, with some side chains indicated in stick form. Note the difference in trajectory of the loop in the Cs^+^-bound structure compared to those with K^+^ and Na^+^ bound. Parallel-eye stereoscopic views are shown.

In our original crystallographic analysis of the *Ct*GEN1 product complex in the presence of Na^+^ ions (18) we did not specify the conformation of the peptide connecting α-helices 9 and 10. In the light of the new structural studies with alternative monovalent ions we re-refined the structure in Na^+^ ions, and observe electron density for this region as a result. The new structure is included with a superposition of the Cs^+^, K^+^ and Na^+^-bound structures shown in Figure 5. While the density does not determine the conformations of all the amino acid side chains, the trajectory of the loop is well defined. The loop adopts the same conformation as that in the presence of K^+^ ions, with an RMSD = 0.36 Å. The loop is again more compacted, with D199 located 12 Å from metal ion M2 in the active site.

### Mutations that affect the H2TH loop-active site interaction lower cleavage rate

The importance of the interaction between the H2TH loop and the active site of *Ct*GEN1 has been examined by mutagenesis. We have made point mutants of the interacting partners, and measured rates of cleavage of junction 3 in the presence of 50 mM Na^+^ or K^+^ ions. Mutation to form either D199A or Y200F (the latter removing the phenolic O of the tyrosine so it can no longer hydrogen bond to E120) leads to a 100-fold reduction in cleavage rate in K^+^ ions (Supplementary Table S2). We have previously shown that an E120A mutant also leads to a 100-fold lower cleavage rate (18). Thus any change in the interactions of the D199, Y200 dipeptide lowers the rate two orders of magnitude. In the presence of Na^+^ ions these rates are lowered a further 5- to 10-fold. This indicates that reducing the stability of the extended conformation of the H2TH loop has a marked effect on the activity of *Ct*GEN1. The double mutant D199A, Y200F leads to almost complete loss of activity of *Ct*GEN1.

Taken together, our crystallographic results indicate that the H2TH loop has a bistable structure that can adopt either of two conformations, and is altered by the nature of the monovalent metal bound at the neck of the loop. The functionally significant difference between the structures is that D199 at the extremity of the loop locates into the active site, or retracts away from it.

## DISCUSSION

As a group of enzymes the FEN/EXO family all cleave branched DNA substrates 1 nt 3′ to the branchpoint, and share a similar core architecture. But the four-way DNA junction substrate is more complex, requires two cleavages for resolution and thus GEN1 must act in dimeric form. We now show here that in the presence of K^+^ ions (the predominant monovalent cation inside eukaryotic cells) the substrate range is broadened a little, and that the simpler branched substrates can bind to *Ct*GEN1. This perhaps shows the familial origins of GEN1, yet even in K^+^ ions the four-way junction is cleaved faster and bound more tightly. GEN1 has specialized to become quite specific for four-way DNA junctions. It is likely that the binding and cleavage of other substrates in the presence of K^+^ ions results from a kind of substrate mimicry, whereby one or two arms might be accommodated on the GEN1 binding surface. We have previously shown that this has an electrostatic complementarity to duplex DNA (18) and would expect that sections of duplex might bind into those sites as has been observed for human GEN1 (19). The three-way junction and nicked three-way junction make an useful comparison, since they differ only in the presence or absence of the strand break. Both bind *Ct*GEN1, yet only the nicked three-way junction is significantly cleaved. The 3H three-way junction is known to be conformationally rigid (41), and the nick will add considerable flexibility to the structure. It is therefore probable that the nicked three-way junction can adopt a structure similar to that of three arms of the four-way junction when bound to *Ct*GEN1 and so be better accommodated into the active site and cleaved at the usual position. The splayed helix species shows the greatest sensitivity to the nature of the monovalent ion, yet it is cleaved much more slowly than the four-way junction probably because it lacks the second duplex arm that binds to the side of *Ct*GEN1 with the chromodomain. Thus it appears likely that the real substrate for cleavage in the cell is the four-way junction, and its main functional role is the resolution of Holliday junctions.

The Holliday junction-resolving enzymes as a class make bilateral cleavages, yet these do not occur simultaneously (34,35). Therefore a critical characteristic of Holliday junction-resolving enzymes is the acceleration of second strand cleavage. We have shown that under optimal conditions in the presence of K^+^ and Mg^2+^ ions this acceleration is substantial, at nearly 17-fold. This is greater than that for the yeast mitochondrial junction-resolving enzyme CCE1 (34), and should ensure productive resolution of the junction in the lifetime of the DNA–enzyme complex.

**Figure 6.**
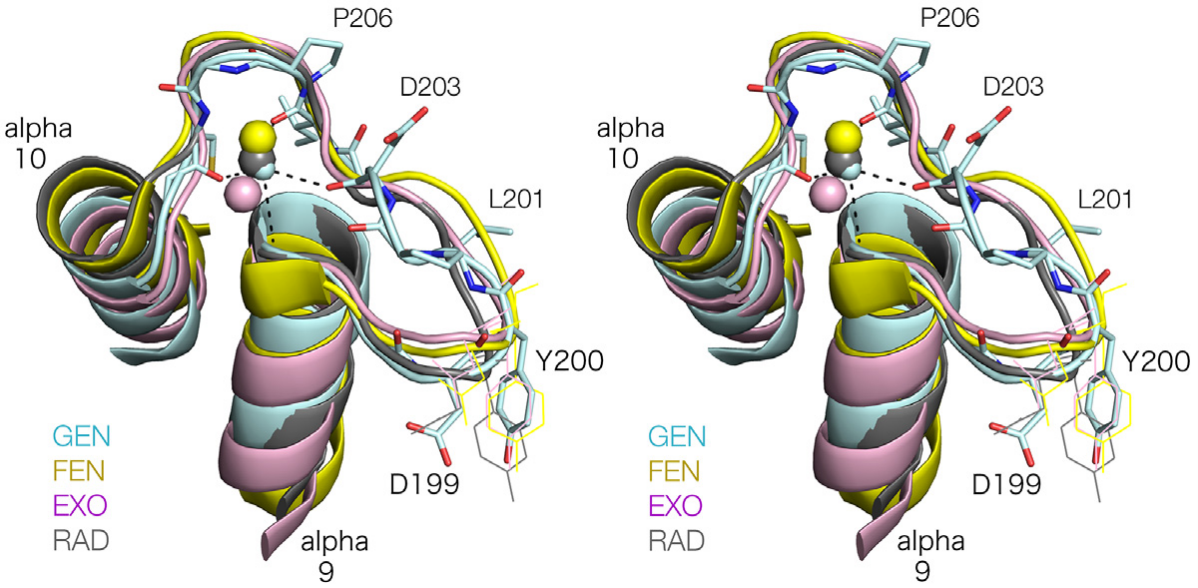
Superposition of the H2TH loops of *Ct*GEN1 (Cs^+^), human FEN1 (K^+;^ 3Q8K), human EXO1 (K^+;^ 3QE9) and *Saccharomyces cerevisiae* Rad2 (K^+;^ 4Q0W). Parallel-eye stereoscopic views of the four H2TH structures in cartoon form, with a ribbon following the trajectory of the loop region. Some amino acid side chains are numbered according to the *Ct*GEN1 numbering scheme.

A key feature of the duplex binding site of the FEN/EXO family enzymes is the H2TH motif that binds a monovalent ion, generally K^+^. It has recently been proposed that FEN1 makes its initial contact with its DNA substrate via the H2TH domain (42). We have now found that *Ct*GEN1 has a closely similar structure, yet its conformation of depends upon the nature of the bound cation. The structure adopted in the crystal by *Ct*GEN1 bound to its DNA product in the presence of Cs^+^ ions is closely similar to the complexes of FEN1, EXO1 and XPG/Rad2 in K^+^ ions (Figure 6). The four H2TH structures adopt very similar conformations, with the universal D199 and Y200 (using the *Ct*GEN1 numbering) placing the carboxylate group of D199 into the active site. This is strongly supported by our mutational analysis, where any single mutations results in a 100-fold impairment of cleavage activity and a double mutant is essentially inactive. The H2TH loops of all four enzymes coordinate the two non-bridging oxygen atoms of the phosphate group 6 nt from the scissile phosphate on the opposite strand in the 5′ direction. This is located on the same side of the DNA, across the major groove from the scissile phosphate, i.e. on the face of the helix interacting with the enzyme surface. The phosphate group immediately 5′ to that coordinated to the ion is located on the axis of α10 at its N-terminus, where it interacts with the positive pole of the helix dipole. The loop trajectory that defines the monovalent ion binding sites is closely similar in the four enzymes, and the coordination of the ions by amino acids within the loop of the H2TH is similar though not identical. Only *Ct*GEN1 uses the backbone oxygen atoms of four amino acids to coordinate the metal ion, and the only interaction that is common to all four is made with the most C-terminal residue corresponding to C208 in *Ct*GEN1.

Our structural analysis of *Ct*GEN1 reveals that the H2TH can adopt one of two alternative conformations (Figure 5). In Na^+^ and K^+^ ions the loop is less extended, so that the D199, Y200 is substantially retracted away from the active site. It is perhaps surprising that in *Ct*GEN1 the extended conformation is only observed in Cs^+^, and not in K^+^ ions. Given the differences we observe for the activity of *Ct*GEN1 in Na^+^ and K^+^ ions we might have expected structural differences between the structures adopted in these two ions, yet they are quite similar. There are two potential reasons for this. First, the K^+^ and Cs^+^ crystals were generated by soaking the Na^+^-bound *Ct*GEN1 crystals, rather than being crystallized in the presence of the respective monovalent ions. Second, our crystals contain *Ct*GEN1 in complex with its product, not an intact junction. It is quite possible that the H2TH changes conformation following the cleavage of its substrate. As a working model we suggest that in the presence of K^+^ ions *Ct*GEN1 binds to the junction with the H2TH in its extended form, with D199 engaged in the active site. On the first cleavage reaction the subunit that catalyzed that reaction then might change into the structure we observe in Na^+^ and K^+^ ions, and this conformational change could be communicated to the second subunit whose cleavage reaction is accelerated as a result. By exploiting this conformational change (together perhaps with a change in the conformation of the DNA junction after the first cleavage) it could effectively ensure bilateral cleavage within the lifetime of the complex. This is critical to the function of the Holliday junction-resolving enzymes.

## DATA AVAILABILITY

Atomic coordinates and structure factor amplitudes have been deposited with the PDB with accession codes 6GRC, 6GRB and 6GRD.

## Supplementary Material

Supplementary Data
