# Supplementary material for: A monovalent ion in the DNA binding interface of the eukaryotic junction-resolving enzyme GEN1
Source: Nucleic Acids Res. 2018 Sep 24;46(20):11089–98. doi: 10.1093/nar/gky863 (PMC6237754; doi:10.1093/nar/gky863)
Supplement: Supplementary Data [file gky863_supplemental_files.pdf]

Monovalent ions in the DNA binding interface of the eukaryotic junction-resolving enzyme GEN-1

Y. Liu, A. D. J. Freeman, A.-C. Déclais and D. M. J. Lilley

SUPPLEMENTARY INFORMATION

SUPPLEMENTARY FIGURES

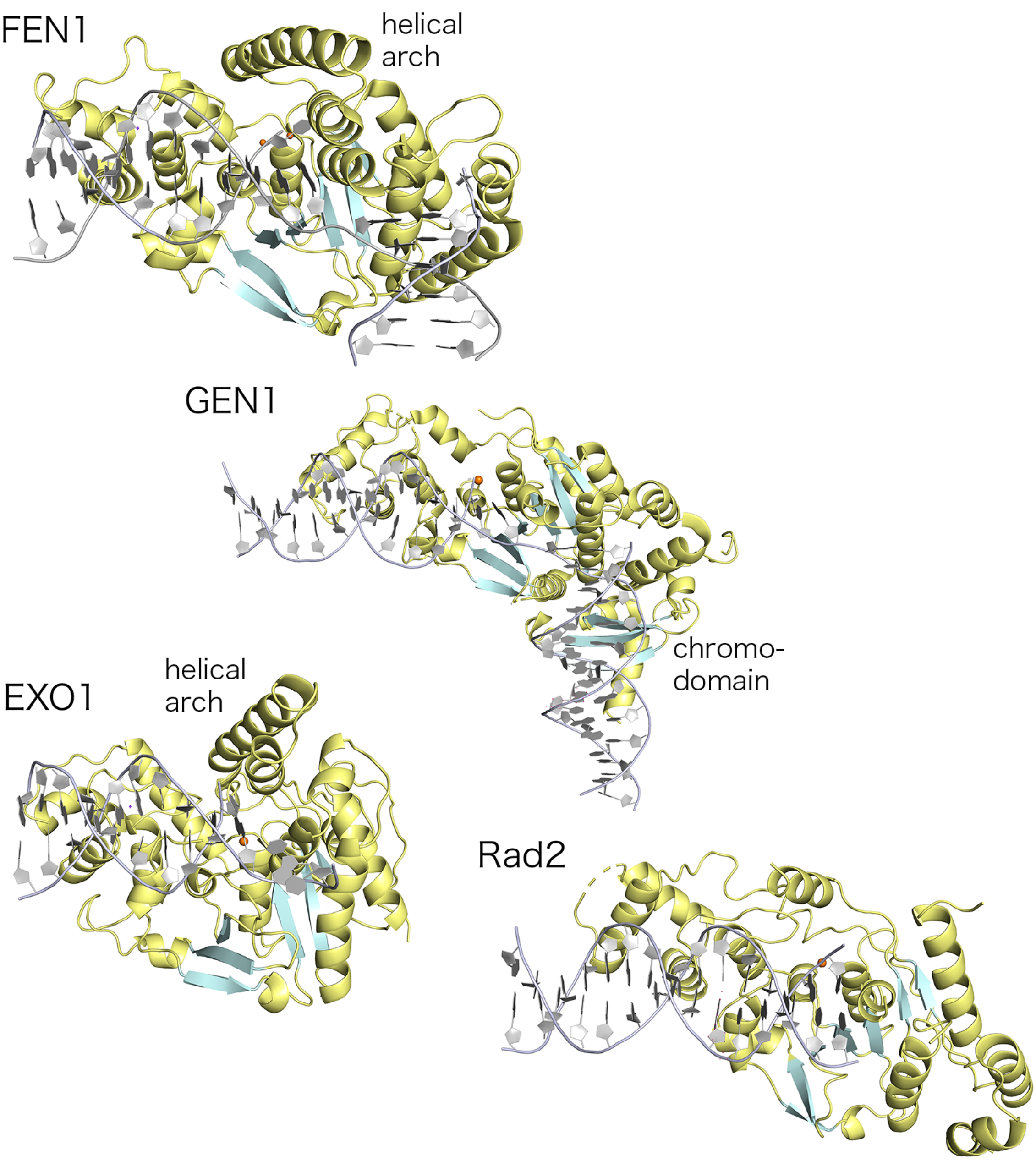

Figure S1. The structures of *Ct*GEN1 (5CNQ) (1), *Hs*FEN1 (3Q8K) (2), *Hs*EXO1 (3QE9) (3) and *Sc*Rad2 (4Q0W) (4) compared. Each is shown as a complex with DNA. GEN1 is shown as the complex of a monomer with one product of resolution. The proteins are shown in cartoon form, with β-sheet colored cyan. The helical arch regions of FEN1 and EXO1 are labeled, as is the C-terminal chromodomain of *Ct*GEN1.

1. Liu, Y., Freeman, A.D.J., Déclais, A.-C., Wilson, T.J., Gartner , A. and Lilley, D.M.J. (2015) Crystal structure of a eukaryotic GEN1 resolving enzyme bound to DNA. *Cell Reports*, **13**, 2565-2575.
2. Tsutakawa, S.E., Classen, S., Chapados, B.R., Arvai, A.S., Finger, L.D., Guenther, G., Tomlinson, C.G., Thompson, P., Sarker, A.H., Shen, B. *et al*. (2011) Human flap endonuclease structures, DNA double-base flipping, and a unified understanding of the FEN1 superfamily. *Cell*, **145**, 198-211.
3. Orans, J., McSweeney, E.A., Iyer, R.R., Hast, M.A., Hellinga, H.W., Modrich, P. and Beese, L.S. (2011) Structures of human exonuclease 1 DNA complexes suggest a unified mechanism for nuclease family. *Cell*, **145**, 212-223.
4. Mietus, M., Nowak, E., Jaciuk, M., Kustosz, P., Studnicka, J. and Nowotny, M. (2014) Crystal structure of the catalytic core of Rad2: insights into the mechanism of substrate binding. *Nucleic Acids Res*., **42**, 10762-10775.

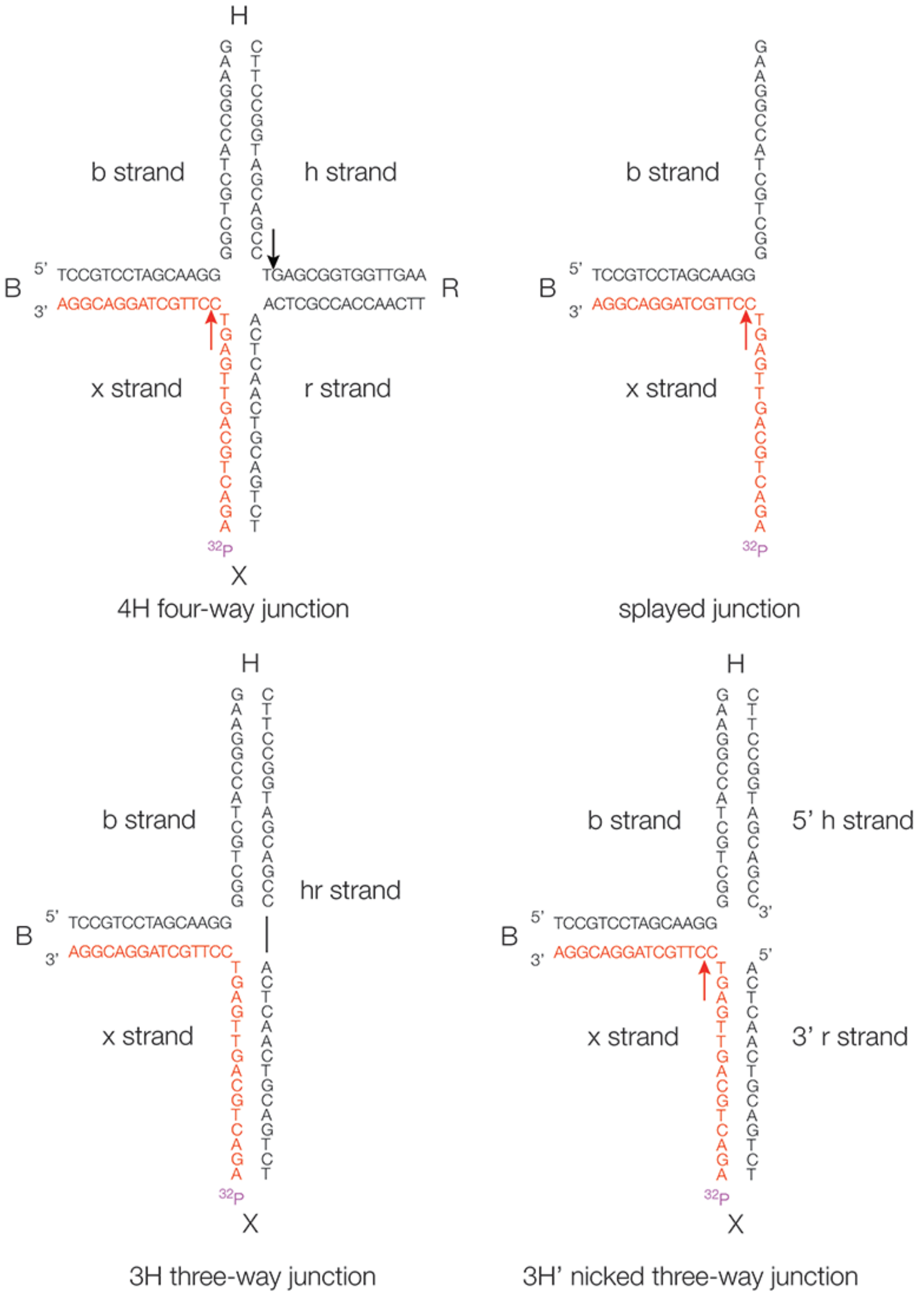

Figure S2. The sequences, secondary structures and cleavage sites of the different branched substrates used to examine cleavage activity by *Ct*GEN1 in the presence of Na^+ and K^+ ions. The common element is the B arm, and the [5'-^{32}P]-labeled x strand (red). In these depictions 10 nt have been removed from each end of the 50 nt long strands, and from the 5' and 3' ends of the 5'h and 3'r strands respectively. The full sequences are shown in Table S1. Cleavage sites (1 nt 3' to the junction) are shown by the red arrows. The second site in the four-way junction is shown black, because this is not detected using the x-strand labelling scheme.

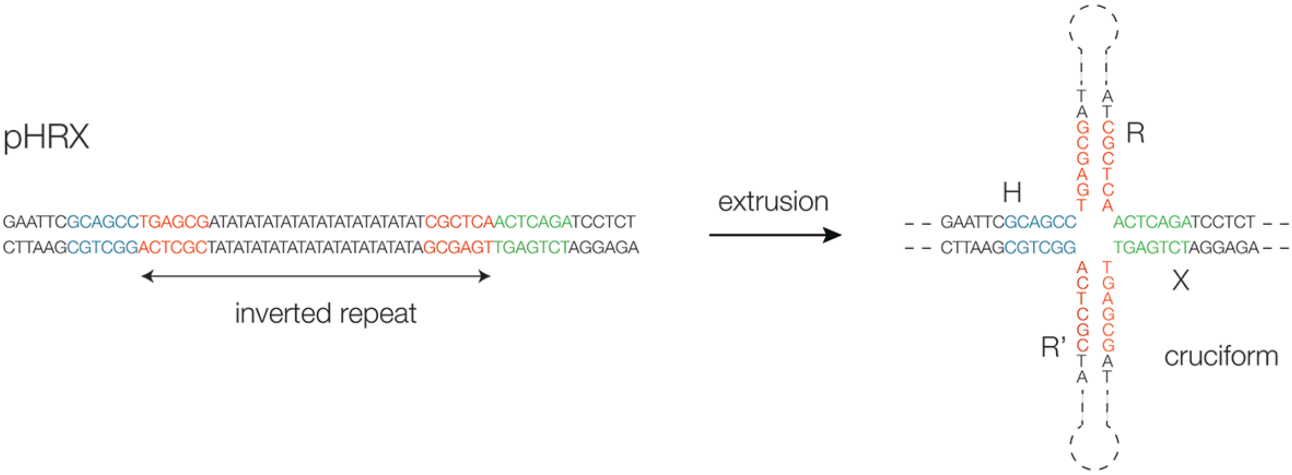

Figure S3. The sequence of the pHRX plasmid cruciform shown as an inverted repeat (left) and as an extruded cruciform structure (right). In its cruciform conformation the four-way junction comprises the H, R and X arms of the standard junction 3. The central 24 bp of the inverted repeat comprises a $(AT)_{12}$ repeat that confers facile extrusion of the cruciform structure in the negatively supercoiled plasmid. This cruciform has been used for the kinetic measurements reported in this work.

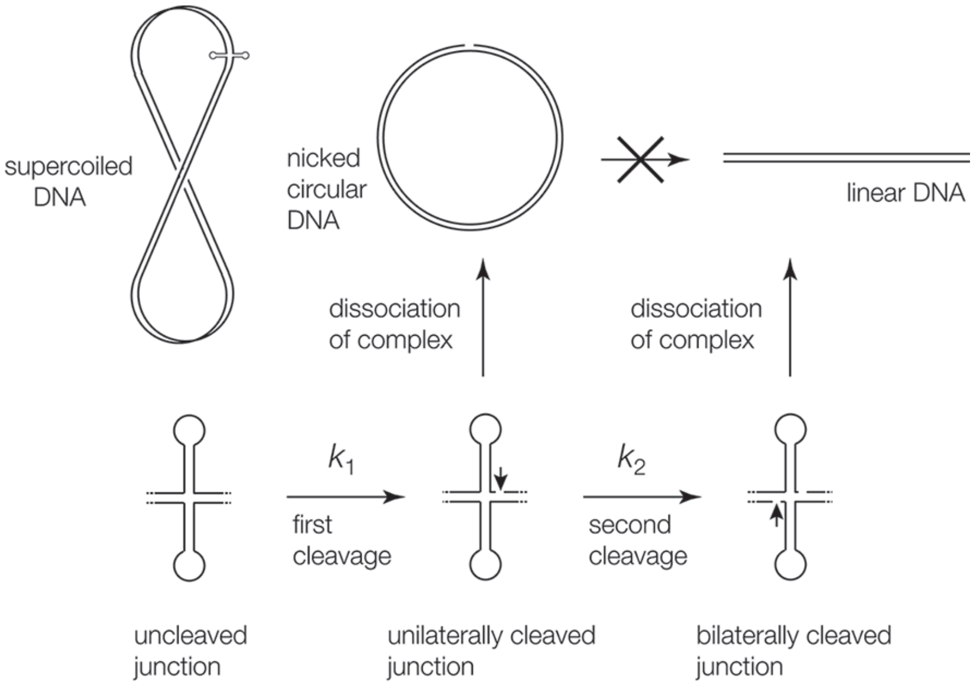

Figure S4. Scheme showing the principle of the cruciform cleavage experiment. The cruciform structure is not stable in non-supercoiled DNA but is stabilized in negatively supercoiled DNA due to its local twist change. Once a cleavage is introduced the cruciform by the action of a nuclease the supercoiling is released and the cruciform is no longer stable and is absorbed into the body of the plasmid. Thus if the resolving enzyme dissociates after a unilateral cleavage, no further cleavage is possible because the substrate no longer exists, and the product is nicked circular DNA. However bilateral cleavage leads to the formation of a linear DNA product. The first cleavage event occurs with rate k_1, and the second with k_2. Supercoiled, nicked and linear DNA can be readily separated by agarose gel electrophoresis.

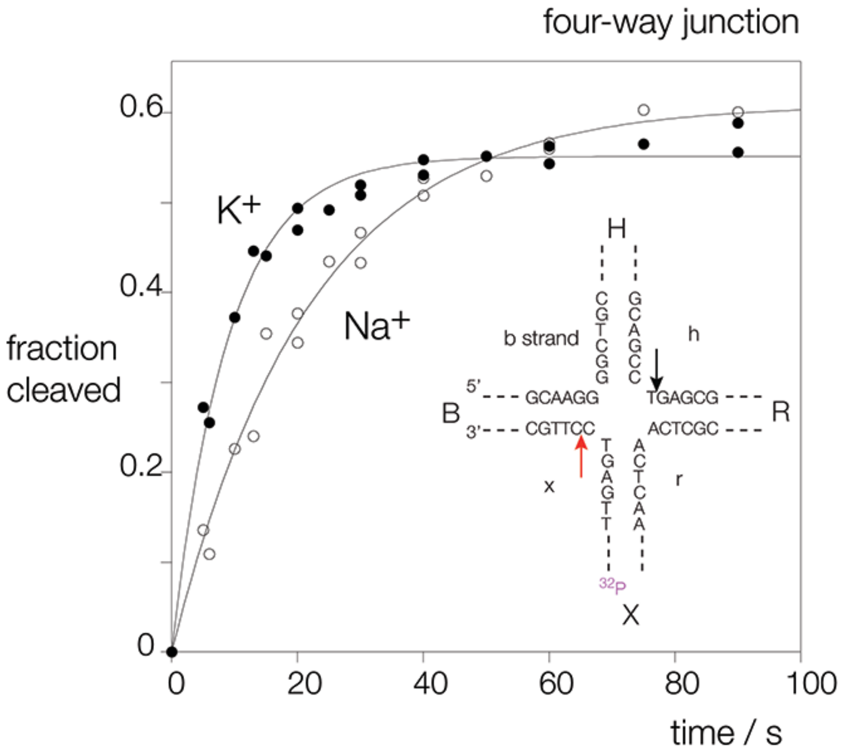

Figure S5. Reaction progress for cleavage of a four-way DNA junction by *Ct*GEN1 as a function of time in the presence of either K^+ (closed circles) or Na^+ ions (open circles). Reactions were performed under single-turnover conditions in the presence of 10 mM cacodylate (pH 6.5), 1 mM $MgCl_2$, 50 mM NaCl or KCl, 0.1% BSA at 37°C.

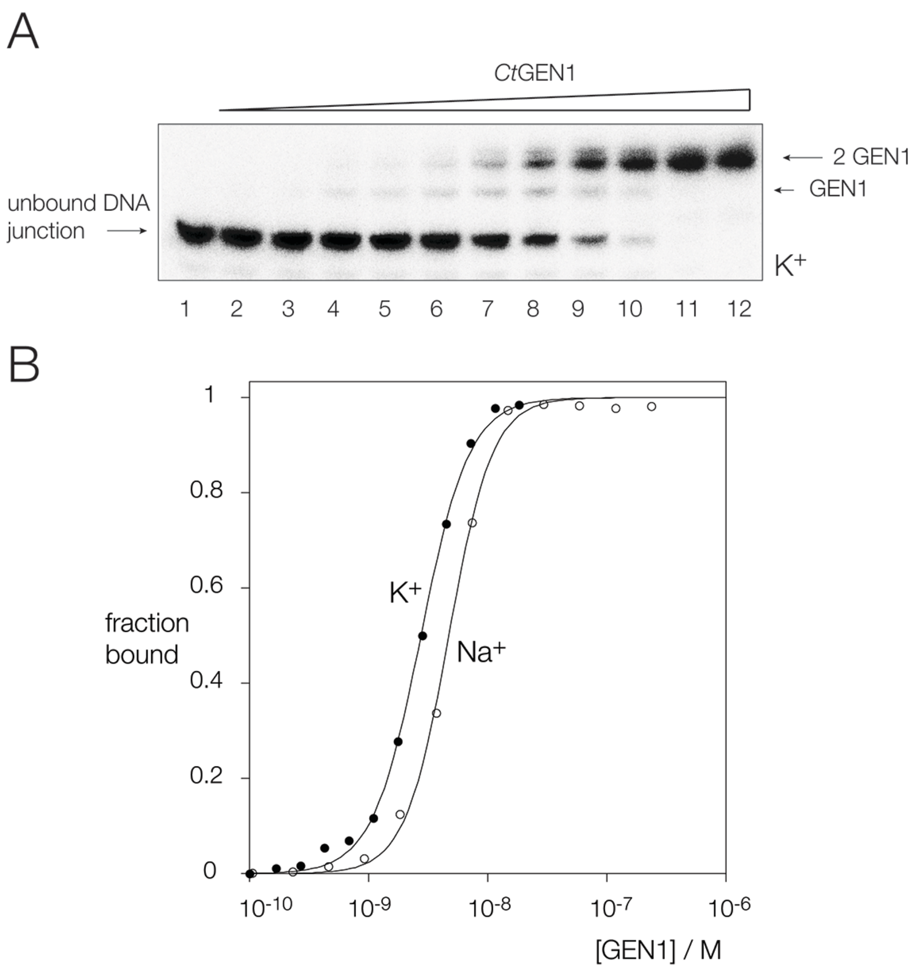

Figure S6. Affinity of *Ct*GEN1 binding to a four-way junction in K^+ and Na^+ ions.

A. 0.1 nM junction was incubated with an increasing concentration of *Ct*GEN1 and electrophoresed in a 6 % polyacrylamide gel in the presence of 50 mM K^+ ions. The concentrations were (tracks 1 – 12) : 0, 0.17, 0.27, 0.43, 0.68, 1.10, 1.75, 2.80, 4.49 and 7.18, 11.48 and 18.38 nM. The major band of *Ct*GEN1-bound junction contains a dimer of *Ct*GEN1 (2 GEN1). At intermediate concentrations a small quantity of bound monomer is visible (GEN1).

B. Binding isotherms for binding in K^+ ions (closed circles) and Na^+ ions (open circles). The data have been fitted to the Hill equation (lines) from which dissociation constants for binding were calculated. Note that the lowest concentration of *Ct*GEN1 is really zero, i.e. corresponds to protein-free DNA junction.

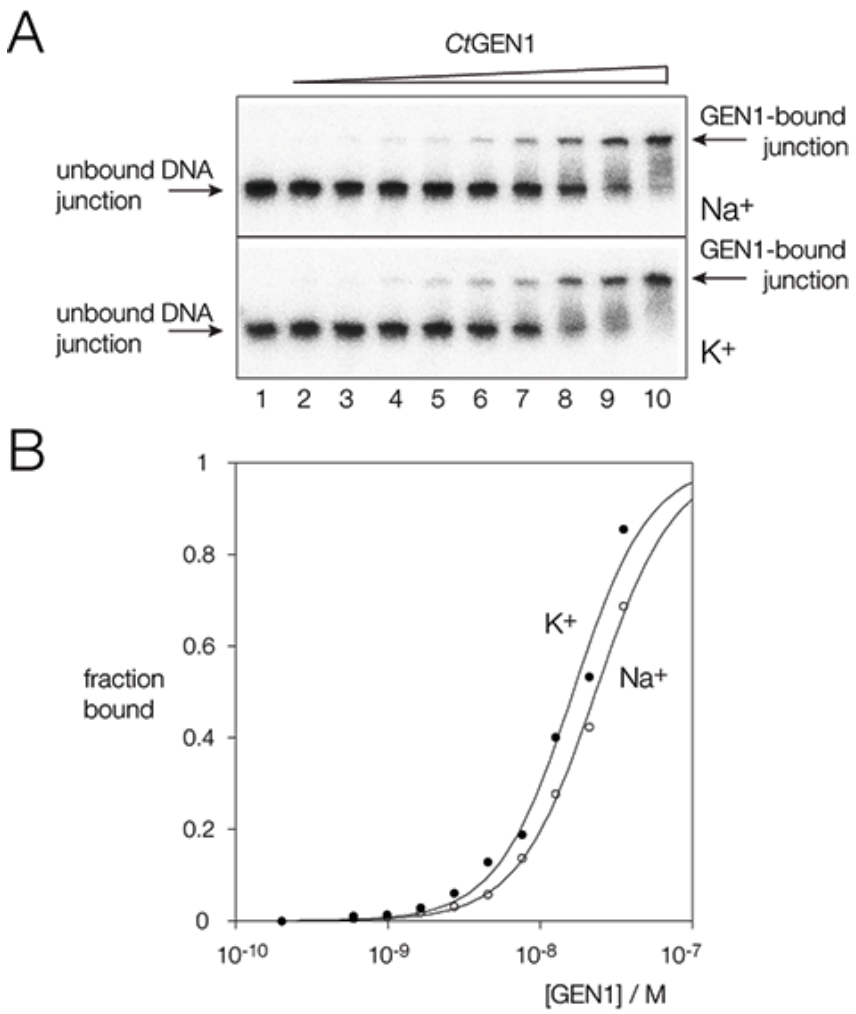

Figure S7. Affinity of *Ct*GEN1 binding to a three-way junction in K^+ and Na^+ ions.

A. 0.5 nM junction was incubated with an increasing concentration of *Ct*GEN1 and electrophoresed in a 6 % polyacrylamide gel in the presence of either 50 mM Na^+ or K^+ ions. The concentrations were (tracks 1 – 10) : 0, 0.59, 0.989, 1.63, 2.72, 4.54, 7.56, 12.6, 21.0 and 35.0 nM.

B. Binding isotherms for binding in K^+ ions (closed circles) and Na^+ ions (open circles). The data have been fitted to the Hill equation (lines) from with dissociation constants for binding were calculated. Note that the lowest concentration of *Ct*GEN1 is really zero, i.e. corresponds to protein-free DNA junction.

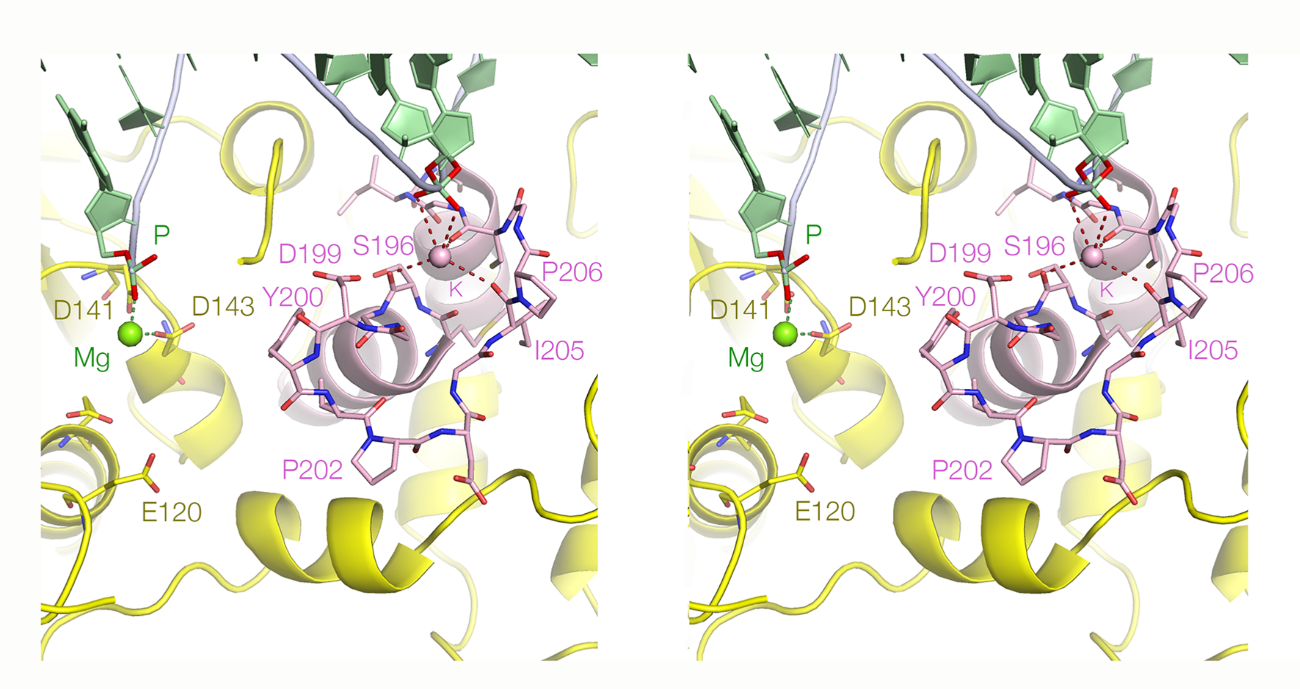

Figure S8. The structure of the H2TH loop of *Ct*GEN1 with a K^+ ion (K) bound. The protein is colored yellow except for the H2TH region that is colored pink. The view shown is similar to that presented in Figure 4A for the structure with Cs^+ bound. The active site and the M2 Mg^{2+} ion (Mg, green) lies on the left in this view. Note that D199, Y200 do not approach the active center in this structure.

SUPPLEMENTARY TABLES

All DNA sequences are written 5' to 3'

Four-way junction 3

All strands 50 nt in length

b-strand CCTCGAGGGATCCGTCCTAGCAAGGGGCTGCTACCGGAAGCTTACAGATG
h-strand CATCTGTAAGCTTCCGGTAGCAGCCTGAGCGGTGGTTGAATTCACAGATG
r-strand CATCTGTGAATTCAACCACCGCTCAACTCAACTGCAGTCTAGAACACATG
x-strand CATGTGTTCTAGACTGCAGTTGAGTCCTTGCTAGGACGGATCCCTCGAGG

Three-way junction (junction 3 B, R and X arms)

All strands 50 nt in length

br-strand CCTCGAGGGATCCGTCCTAGCAAGGACTCAACTGCAGTCTAGAACACATG
r-strand CATCTGTGAATTCAACCACCGCTCAACTCAACTGCAGTCTAGAACACATG
x-strand CATGTGTTCTAGACTGCAGTTGAGTCCTTGCTAGGACGGATCCCTCGAGG

Nicked three-way junction (as three-way junction, but with central nick on br strand)

Strands 50 or 25 nt in length

b-strand CCTCGAGGGATCCGTCCTAGCAAGGGGCTGCTACCGGAAGCTTACAGATG
x-strand CATGTGTTCTAGACTGCAGTTGAGTCCTTGCTAGGACGGATCCCTCGAGG
h-strand 1-25 CATCTGTAAGCTTCCGGTAGCAGCC
r-strand 26-50 ACTCAACTGCAGTCTAGAACACATG

Splayed arm junction

Strands 50 nt in length

b-strand CCTCGAGGGATCCGTCCTAGCAAGGGGCTGCTACCGGAAGCTTACAGATG
x-strand CATGTGTTCTAGACTGCAGTTGAGTCCTTGCTAGGACGGATCCCTCGAGG

Table S1. Sequences of oligonucleotides used to construct the various DNA junctions used to analyze *Ct*GEN1 substrate specificity in Na^{+} and K^{+} ions.

DNA	*Ct*GEN1	rate / s^{-1}				K_d / nM (n)	
		Na^+	K^+	Rb^+	Cs^+	Na^+	K^+
4H	wt	4.5×10^{-2}	1.1×10^{-1}	1.0×10^{-1}	5.8×10^{-2}	4.7 (2.4)	2.7 (2.1)
3H	wt	$<1 \times 10^{-4}$	2.9×10^{-4}			23 (1.7)	17 (1.7)
3H nick	wt	7×10^{-3}	5.7×10^{-2}			31 (1.5)	30 (1.2)
splayed	wt	3.8×10^{-5}	6×10^{-4}			93 (2.2)	56 (1.1)
4H	D199A	3×10^{-4}	1.1×10^{-3}				
4H	Y200F	1.7×10^{-4}	1×10^{-3}				
4H	D199A Y200F	1.5×10^{-5}	2×10^{-5}				

Table S2. Rates and binding affinities for *Ct*GEN1. Rates of cleavage and dissociation constants have been measured for the four-way DNA junction 3. Rates were determined under single-turnover conditions in the presence of Na^+ or K^+ ions for the branched species shown in Figure S1. Rates of cleavage were measured in the presence of Rb^+ or Cs^+ ions for the four-way junction only. Cleavage rates were also measured in the presence of Na^+ or K^+ ions for the four-way junction using single and double mutants of *Ct*GEN1. Dissociation constants for wild-type *Ct*GEN1 were measured by electrophoretic retardation analysis in the presence of Na^+ or K^+ ions for the four branched species. Data were fitted to the Hill equation, and the Hill coefficients are reported in parenthesis.

	*Ct*GEN1-Na^{+}	*Ct*GEN1-K^{+}	*Ct*GEN1-Cs^{+}
**Data collection**			
Wavelength (Å)	0.91376	0.9750	1.2000
Resolution range (Å)	48.99 - 2.452 (2.54 - 2.452)	69.23 - 2.40 (2.49 - 2.40)	68.81-2.66 (2.73 – 2.66)
Space group	P $3_1$21	P $3_1$21	P $3_1$21
Unit cell	98.43, 98.43,119.73; 90, 90, 120	97.96, 97.96,119.76; 90, 90, 120	97.39,97.39,119.90; 90,90,120
Total reflections	504825 (45029)	169872 (24958)	379994 (28491)
Unique reflections	25096 (2405)	26441 (3806)	19265(1406)
Multiplicity	20.1 (18.8)	6.4 (6.6)	19.7 (20.3)
Completeness (%)	99.9 (99.5)	99.80 (100.00)	99.99 (99.99)
Mean I/σ (I)	16.3 (1.2)	5.8 (1.0)	15.0 (1.3)
Wilson B-factor	59.3	63.42	69.7
R_{merge}	0.124 (2.518)	0.136 (1.502)	0.150 (2.608)
$CC_{1/2}$	0.999 (0.532)	0.992 (0.211)	0.998 (0.558)
**Refinement**			
R-work	0.2298 (0.3691)	0.2466 (0.3602)	0.2444 (0.3859)
R-free	0.2588 (0.3636)	0.2730 (0.3780)	0.2624 (0.4578)
Number of atoms			
macromolecules	3804	3582	3686
ions	1	2	2
water	37	8	
rmsd			
bond lengths (Å)	0.012	0.009	0.0012
bond angles (°)	1.47	1.1	1.31
Average B-factor	76.69	76.06	77.34
macromolecules	76.72	76.09	77.34
ion	75.18	70.46	79.06
Water	73.43	60.96	
PDB	6GRC	6GRB	6GRD

Table S3. Details of data collection and refinement statistics for the data as deposited in the PDB. Statistics for the highest resolution shell are in parenthesis.
